# Diabetes Mellitus in Outpatients in Debre Berhan Referral Hospital, Ethiopia

**DOI:** 10.1155/2016/3571368

**Published:** 2016-01-06

**Authors:** Tesfa Dejenie Habtewold, Wendwesen Dibekulu Tsega, Bayu Yihun Wale

**Affiliations:** ^1^College of Health Science, Department of Nursing, Debre Berhan University, 445 Debre Berhan, Ethiopia; ^2^College of Health Science, Department of Public Health, Debre Berhan University, 445 Debre Berhan, Ethiopia

## Abstract

*Introduction*. Diabetes mellitus is a group of metabolic diseases characterized by hyperglycemia resulting from defects in insulin secretion, insulin action, or both. Most people with diabetes live in low- and middle-income countries and these will experience the greatest increase in cases of diabetes over the next 22 years.* Objective*. To assess the prevalence and associated factors of diabetes mellitus among outpatients of Debre Berhan Referral Hospital.* Methods and Materials*. A cross-sectional study was conducted from April to June 2015 among 385 patients. Random quota sampling technique was used to get individual patients and risk factors assessment. Patients diabetes status was ascertained by World Health Organization Diabetes Mellitus Diagnostic Criteria. The collected data were entered, cleaned, and analyzed and Chi-square test was applied to test any association between dependent and independent variable.* Result*. Out of the total 385 study patients, 368 have participated in the study yielding a response rate of 95.3%. Concerning clinical presentation of diabetes mellitus, 13.3% of patients reported thirst, 14.4% of patients declared polyurea, and 14.9% of patients ascertained unexplained weight loss. The statistically significant associated factors of diabetes mellitus were hypertensive history, obesity, the number of parities, and smoking history.* Conclusion*. The prevalence of diabetes mellitus among outpatients in Debre Berhan Referral Hospital was 0.34% and several clinical and behavioral factors contribute to the occurrence of diabetes mellitus which impose initiation of preventive, promotive, and curative strategies.

## 1. Introduction

Diabetes mellitus is a group of metabolic diseases characterized by chronic hyperglycemia resulting from defects in insulin secretion, insulin action, or both. It is classified as type 1 diabetes, type 2 diabetes, gestational diabetes, and other types of diabetes mellitus [1]. Diabetes mellitus is the most common chronic disease among adults. The global burden of diabetes has increased twelvefold between 1985 and 2011 [2].

In 2013, 382 million people had diabetes; this number is expected to rise to 592 million by 2035. Most people with diabetes live in low- and middle-income countries and these will experience the greatest increase in cases of diabetes over the next 22 years [3, 4]. According to the international diabetes federation 2013 reports, in North America and Caribbean countries 1 in 10 adults has diabetes; in Southern and Western America 1 in 11 adults has diabetes. Similarly, in Europe 21-22 million people have diabetes [5]. Moreover, in 2013, the number of people with diabetes is estimated to be 56 million in Europe with an overall estimated prevalence of 8.5%. However, estimates of diabetes prevalence in 2013 vary widely in the 56 diverse countries in Europe from 2.4% in Moldova to 14.9% in Turkey [6].

On the contrary in 2010, 12.1 million people were to be living with diabetes mellitus in Africa and over the next 20 years the number of people with diabetes will almost double [2, 7, 8]. Based on the IDF Diabetes Atlas 2014 update the age-standardized prevalence of diabetes in the Middle East and North Africa was estimated at 10.9% and projected to increase to 11.3% by 2035 [9]. Additionally, a systematic review by Bos and Agyemang revealed that the prevalence of diabetes varied across Northern African countries ranging from 2.6% in rural Sudan to 20% in urban Egypt. Ten studies distinguished between urban and rural diabetes prevalence and all of these studies found a higher prevalence in urban areas than in rural areas [10].

According to 2011 reports of the International Diabetes Federation (IDF), the number of adults living with diabetes in Ethiopia was 3.5% [11]. Even though the national prevalence of diabetes in Ethiopia is estimated to be 2%, evidence suggests that its prevalence could be more than 5% in those older than 40 years of age in some setting [12–14]. A study by Watkins and Alemu conducted in Gondar found out most of the rural patients (77%) had type 1 diabetes whereas in urban areas only 29% had type 1 and 71% of them type 2 diabetes [15]. Generally, the global burden of diabetes mellitus has been increasing radically. The impact is high especially in developing countries in which resource is limited to identify the problem and develop need based clinical and community intervention. Therefore, the objective of this study was to assess the prevalence and associated factors of diabetes mellitus among outpatients of Debre Berhan Referral Hospital.

## 2. Methods and Materials

### 2.1. Study Setting

Debre Berhan is the capital city of North Shoa, one of the 13 zones of Amhara regional state which is located 130 KM north of Addis Ababa, Ethiopia. The foundation of the town was traced back to the regime of Atse Zereyakob. Regarding health services in the city, there are one government and one private hospital, two government health centers, five health posts, and 18 private clinics. Debre Berhan Referral Hospital is the only government hospital in the city and it is zonal referral hospital serving the population of the zone as a referral center and the place where this study was conducted [16].

### 2.2. Study Design and Population

A cross-sectional study was conducted from April to June 2015 among 385 patients who visited the outpatient department of Debre Berhan Referral Hospital. All outpatients who visited the hospital during the data collection period were included. Nevertheless, patients who were severely ill, not cooperative, having difficulty in hearing, and visual impairment were excluded. The hospital has many units organized to render care for clients. From these units outpatient units 1, 2, 3, and 5, dental clinic, pediatrics outpatient unit 1, and maternal health unit were selected using simple random sampling technique. To reach individual patients, random quota sampling technique was used.

### 2.3. Data Collection Tools and Procedures

The questionnaire has three parts: sociodemographic characteristics, WHO Diabetes Mellitus Diagnostic Criteria [17], and associated risk factors assessment. The patients' diabetes status was ascertained by considering two classic clinical symptoms and laboratory test of random blood glucose level. To classify diabetes mellitus into type 1, type 2, and gestational, classic symptoms and signs, the age of the patient, random blood sugar level, and pregnancy status were used as a criterion. The data were collected by internship nursing students and professional nurses in selected unit using pretested, structured interviewer administered questionnaire. Also, the standard “forward-backward” procedure was applied to translate the questionnaire from English into Amharic. To ensure data quality, orientation was given for all patients, data collectors were trained, and appropriate study design and sampling technique were deliberated. Additionally, a pretest was done on 5% of respondents. The data was entered, cleaned, and analyzed. Chi-square test was applied to test any association between dependent and independent variable using significance level (*α*) 0.05. To calculate the exact *p* value, Social Science Statistics *p* value calculator was used [18]. Fisher's exact test was also used when the chi-square test assumption was not fulfilled. Finally, the result was presented using descriptive statement, table, and figure.

### 2.4. Ethical Consideration

This study was done in conformity with the ethical guidelines approved by the Institute of Medicine and Health Science of Debre Berhan University. By explaining objectives of the study and its significance, relevant permission was obtained from hospital administration office. At individual level verbal consent was obtained from all patients.

## 3. Result

### 3.1. Sociodemographic Characteristics

Out of the total 385 study participants, 368 have participated in the study yielding a response rate of 95.32%. As described in Table 1, among the patients more than half (53.26%) of them were females. The majority of respondents (30.98%) were in the age group of <30. Additionally, most of the study subjects (74.45%) were Amhara and 70.10% were married. Moreover, 27.44% of the patients were illiterate.

### 3.2. Diagnostic Criteria of Diabetes Mellitus

As shown in Table 2, 13.32% of patients reported polydipsia, 14.40% of patients declared polyuria, and 14.94% of patients reported unexplained weight loss. Similarly, 7.07% of the patients had random blood sugar ≥200 mg/dL. Based on these criteria the overall prevalence of diabetes mellitus in Debre Berhan Referral Hospital was 0.34%.

### 3.3. Factors Associated with Diabetes Mellitus

As revealed in Table 3, 6.52% of the respondents had a family history of diabetes mellitus, 2.72% were twins, and 5.43% had previously known hypertensive disease. Also, most of (82.07%) patients did not do regular physical exercise.

**Figure 1:**
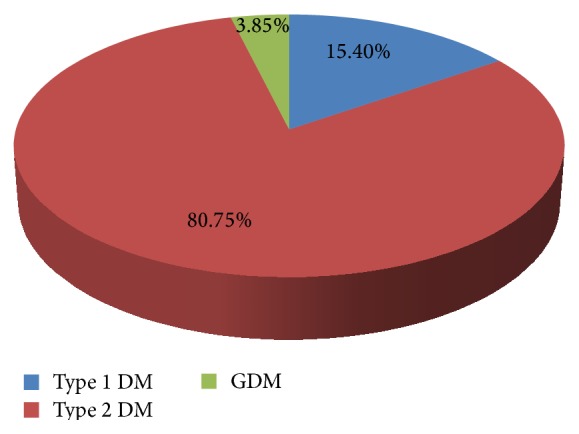
Diabetes mellitus category of patients in Debre Berhan Referral Hospital in June 2015.

As shown in Figure 1, among those diabetes cases, 4 cases (15.4%) were diagnosed as type 1 diabetes mellitus, 80.77% were type 2 DM, 3.85% were gestational type of diabetes mellitus.

**Figure 2:**
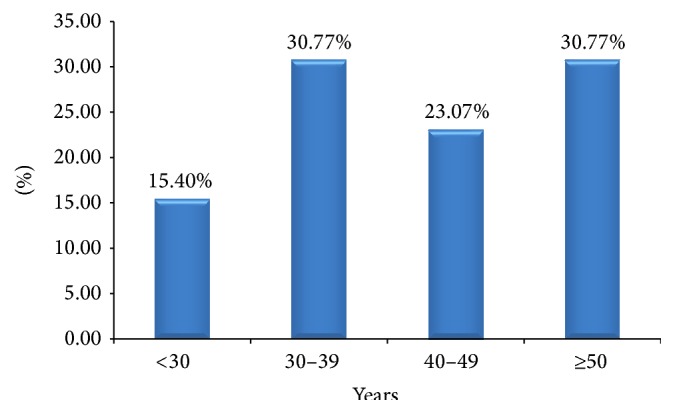
Distribution of diabetes mellitus among different age groups of patients in Debre Berhan Referral Hospital in June 2015.

As portrayed in Figure 2, 15.4% of the diabetes cases were found in the age group of <30; 30.77% of the diabetes cases were found in the age group of 30–39; the other 23.07% of them were found in the age group of 40–49; and about 30.77% of the diabetes cases were found in the age group of ≥50.

Concerning nonclassical symptoms and signs, 15.4% had reported a loss of consciousness, 46.15% reported developing numbness and tingling sensation, 42.3% have blurred vision, and the other 15.4% have reported wounds that cannot heal easily. Furthermore, among the diabetes cases, 23% of them had a history of hypertension.

### 3.4. Statistical Test

As observed from Table 4, the *p* value of family history of diabetes mellitus and twin delivery was greater than 0.05, consequently interpreted as there is no association of family history of diabetes mellitus and twin delivery with the occurrence of diabetes mellitus. On the other hand, hypertensive history, obesity, number of parities, and smoking history have direct association with the occurrence of diabetes mellitus. Among those associated factors hypertensive history has the highest contribution following the number of parities and obesity.

## 4. Discussion

To our knowledge, this study was the first in Debre Berhan. It was conducted with the aim of assessing the prevalence and associated factors of diabetes mellitus.

In this study the percentage of diabetes mellitus among children ≤ 14 years was about 3.85%. Differently in a study by the World Health Organization's multinational project for childhood initially reported in 2000 the prevalence was 19,164 cases from the population of 75.1 million people which are about 0.025% [19]. This difference might be due to variation in sample size. Also, in this study the percentage of type 1 diabetes mellitus was about 15.4%; however, a decreased prevalence of 4% of type 1 diabetes mellitus was observed in the population studied in Asia, about 3.2% in Europe and 5.3% in North America [20]. This might be due to the lifestyle difference between Ethiopia and Western countries.

Furthermore, we found out the frequency of type 1 diabetes was not high in the youngest age group (0–4 years) and the percentage increased (15.4%) after puberty and young adulthood (15–29 years). On the contrary, a study done in Europe suggests that the prevalence rate of type 1 DM was highest in the youngest age group (0–4 years) and prevalence rates decline after puberty and appear to stabilize in young adulthood (15–29 years) [21]. This might be due to the difference in feeding habit, knowledge and health seeking behavior, and living standard.

In addition, in this study the percentage of type 2 diabetes mellitus was 80.77% but a ten-year observation at Gondar University Teaching Referral Hospital found out 49.9% were type 2 DM [22] and the prevalence study in Jimma University stated that 66.2% of the respondents were medically diagnosed as having type 2 diabetes mellitus [23]. This difference might be due to the difference in the duration of time of the study to conclude for the general population.

Concerning associated factors, numerous epidemiological studies were conducted to discriminate the different associated factors. In this study, there is no significant association between family history of diabetes mellitus and the occurrence of diabetes mellitus but a study done on the Palestinians, Iranians, and Kuwaitis documented that family history of diabetes increased the risk of the incidence by 1.6, 1.8, and 2.4 times, respectively [24–26]. In our study, however, the increased body mass index was also one of significant risk factors. This finding was consistent with WHO STEPS report [27], the study done in Israel [28] and Iran and Jordan [29]. Moreover, smoking history was a significant risk factor. This finding was in line with the study conducted in European countries [6]. Other significant risk factors of diabetes mellitus, not assessed in this study (but future researchers should consider them), are elevated triglycerides, total cholesterol, and low HDL cholesterol [30], gender and educational status [24], socioeconomic status [31], and physical inactivity [6, 27, 32, 33].

## 5. Strength and Limitation

The strengths of this study include a high response rate and the inclusive nature of this research as individuals could participate regardless of their demographic variation. Additionally, a reasonable sample size and culturally adapted questionnaires were used. Since it was the first study in Debre Berhan, it will provide basic information for those who have an interest. Furthermore, objective laboratory data were used to ascertain disease status of patients.

Despite these strengths, this study contains the following limitations: since the study was institutional and conducted among outpatients in only one hospital it could limit our understanding regarding the prevalence and associated factors of diabetes mellitus in the setting. Even though data collectors were blind for the study subjects, there might be selection bias. Moreover, due to cross-sectional nature of the study causal relationships between the risk factors and disease outcome could not be assumed. Furthermore, the data was analyzed manually and chi-square model which was a weak measure of association was utilized.

## 6. Conclusion

Diabetes mellitus and other noncommunicable diseases are becoming abundant in developing countries including Ethiopia due to lack of problem identification and intervention of these problems.

This study is targeted to know the prevalence of diabetes mellitus among outpatients in Debre Berhan Referral Hospital and associated factors that contribute to the occurrence of diabetes mellitus accompanied by initiation of preventive, promotive, and curative strategies.

Moreover, the study will help to initiate the community, health institution, and other concerned nongovernmental organizations to give emphasis to the population for controlling of diabetes mellitus. It will also give baseline information for those who aim to conduct a community-based longitudinal research in this area. Finally, mass media, zonal health office, and the Ministry of Health should work on the use of evidence-based medicine and awareness creation by developing an up-to-date guideline tailored to each specific group of the population.

## Figures and Tables

**Table 1:** Sociodemographic characteristics of patients in Debre Berhan Referral Hospital in June 2015.

Variables	Categories	Frequency	Percentage
Sex	Male	172	46.74
	Female	196	53.26

Age	<30	114	30.98
	30–39	112	30.43
	40–49	77	20.92
	>50	65	17.67

Ethnicity	Amhara	274	74.45
	Tigray	24	6.53
	Oromia	66	17.93
	Other	4	1.09

Religion	Orthodox	271	73.64
	Muslim	63	17.12
	Catholic	6	1.63
	Protestant	28	7.61

Marital status	Married	258	70.10
	Single	72	19.56
	Divorced	16	4.35
	Widowed	22	5.99

Educational status	Illiterate	100	27.17
	Can read and write	76	20.65
	Grade 1 to grade 8	63	17.12
	Grade 9 to grade 12	54	14.67
	Diploma and above	75	20.65

Occupational status	Government employed	76	20.65
	Self-employed	90	24.45
	Merchant	42	11.41
	Housewife	87	23.64
	Others	73	19.84

Monthly income status	≤650	189	51.36
	651–1400	75	20.38
	≥1401	104	28.26

**Table 2:** WHO diagnostic criteria to know diabetes mellitus status of patients in Debre Berhan Referral Hospital in June 2015.

Variables	Frequency	Percentage
Classic symptoms		
Thirst		
Yes	49	13.32
No	319	86.68
Polyuria		
Yes	53	14.40
No	311	84.51
Unexplained weight loss		
Yes	55	14.94
No	313	85.06
Classic sign		
RBS ≥ 200 mg/dL		
Yes	26	7.07
No	342	92.93
Others		
Fatigue		
Yes	161	43.75
No	207	56.25
Nausea and vomiting		
Yes	84	22.83
No	284	77.59
Polyphagia		
Yes	23	6.25
No	345	93.75
Headache		
Yes	148	40.22
No	220	59.78
Loss of consciousness		
Yes	15	4.08
No	353	95.92
Numbness and tingling sensation		
Yes	45	12.23
No	323	87.77
Blurring of vision		
Yes	34	9.24
No	334	90.76

**Table 3:** Associated factors among patients in Debre Berhan Referral Hospital in June 2015.

Variables	Frequency	Percentage
Family history of diabetes mellitus		
Yes	24	6.52
No	344	93.48
Twin delivery		
Identical	5	1.36
Fraternal	5	1.36
Previously hypertensive disease		
Yes	20	5.43
No	348	94.57
Activity and exercise		
Good	302	82.07
Poor	66	17.03
Obesity (BMI)		
≤24.9 kg/m^2^	340	92.39
>24.9 kg/m^2^	28	7.61
Number of children delivered		
<2 times	71	19.29
≥2 times	61	16.58
No	33	8.97

**Table 4:** Statistical test for associated factors of diabetes mellitus in Debre Berhan Referral Hospital in June 2015.

Variables	Calculated chi-square value	Degree of freedom	Odds ratio	*p* value
Family history of diabetes mellitus	3.6	1	2.9	0.06
Previous history of hypertension	25.25	1	9.3	**0.000**
Body mass index	9.33	1	4.36	**0.002**
Parity	11.34	1	8.95	**0.0008**
Twin delivery	0.2	1	1.46	0.65
Smoking history	5.45	1	4.33	**0.02**

## Abbreviations

BMI: Body mass index
CBE: Community-based education
DBRH: Debre Berhan Referral Hospital
DBU: Debre Berhan University
DM: Diabetes mellitus
ETB: Ethiopian birr
GDM: Gestational diabetes mellitus
IDDM: Insulin dependent diabetes mellitus
IDF: International diabetes federation
OPD: Outpatient department
PI: Principal investigator
SSB: Sugar sweetened beverage
T_1_ DM: Type 1 diabetes mellitus
T_2_ DM: Type 2 diabetes mellitus
UKDPS: United Kingdom diabetes prospective study
WHO: World Health Organization.
